# Introduction to the Special Issue on "Analysing the Politics of Health Policy Change in Low- and Middle-Income Countries: The HPA Fellowship Programme 2017-2019"

**DOI:** 10.34172/ijhpm.2021.43

**Published:** 2021-04-26

**Authors:** Lucy Gilson, Zubin Cyrus Shroff, Maylene Shung-King

**Affiliations:** ^1^Health Policy and Systems Division, School of Public Health and Family Medicine, University of Cape Town, Cape Town, South Africa.; ^2^Department of Global Health and Development, London School of Hygiene and Tropical Medicine, London, UK.; ^3^The Alliance for Health Policy and Systems Research, World Health Organization, Geneva, Switzerland.

**Keywords:** Health Policy Analysis, Policy Process, Actor-Centric Analysis

## Abstract

This special issue presents a set of seven Health Policy Analysis (HPA) papers that offer new perspectives on health policy decision-making and implementation. They present primary empirical work from four countries in Asia and Africa, as well as reviews of literature about a wider range of low- and middle-income country (LMIC) experience.

## Background

An integral element of the broader field of Health Policy and Systems Research (HPSR), Health Policy Analysis (HPA) seeks to understand how policies emerge, are formed and are implemented, with specific concern for the forces influencing decision-making: actors, power and politics; institutions, interests and ideas.^1,2^ The policy process orientation of HPA draws from the theories and ideas of well-established fields in higher income country settings - policy studies and public administration, as well as political and organisational sciences. At the same time, HPA draws on ideas about the political economy of development in its applications within low- and middle-income countries (LMICs).^3^

HPA supports deeper understanding of the world around us, including how policies support change within health systems, or are resisted or subverted.^3^ Prospectively, HPA can also support policy-makers, advocacy groups and researchers in navigating or seeking to influence health system change.^4^ Nonetheless, HPA has been relatively neglected in LMIC work – as reflected in funding and publications – compared to research on or about health systems.^1,5^

Given its value for health system development, the Alliance for Health Policy and Systems Research, World Health Organization (WHO), initiated the HPA Fellowship programme in 2017. Now supporting its second cohort, a total of 20 fellows registered for doctoral-level studies in higher education institutions in a range of African and Asian countries have received HPA mentorship through the programme. This special issue presents work from the first cohort of Fellows.

The Fellowship’s focus on supporting HPA work conducted *in* LMICs and *by* LMIC researchers who are supported *from* LMIC institutional bases, is a purposeful step towards expanding and deepening LMIC capacity in this area. Nurturing such capacity is part of the decolonial agenda within and outside global health. First, LMIC contexts differ from those of higher income country settings in terms of their histories and colonial legacies, political systems, cultures and practices, government capacities, and donor influence. Second, researchers working within a country are exposed on a daily basis to the political, economic and social realities of that setting and to the processes and personalities influencing policies. They commonly have a deeper awareness of the history of the health system and understanding of its lived realities than external researchers who visit for short periods. They are likely, then, to ask different questions to those from outside that setting, as well as to offer different interpretations of experience. They are also better placed to develop the long-term relationships needed to support this work. Third, and linked, whilst theoretical frames derived from experience in high income settings offer important lenses for inquiry into policy process phenomena, it is also critical to draw on relevant theoretical work derived from LMIC experiences. Cross-disciplinary relationships within countries, and with policy actors, may assist in deepening theoretical reflection and generating new theoretical insights. Fourth, new HPA research and theory must be developed by LMIC analysts working within LMICs in order to become infused into the ecosystem of knowledge and decision-making, as well as to continue to nurture future generations of analysts.

## The Fellowship Programme

In practical terms, the Fellowship programme is coordinated by the University of Cape Town, South Africa, which was selected through a competitive process, and draws on a mentor pool of experienced health policy analysts. As part of its design, supervisors have also been engaged in the programme – to ensure alignment of advice and provide opportunities for their engagement with other HPA work.

The Fellowship brings personal, professional and financial support to the selected candidates. The personal support comes from joining a group of peers working towards doctoral-level qualifications in diverse LMIC settings who learn together, provide support to each other and work collectively in activities like writing blogs and making conference presentations. Professional support is provided in the form of workshops that provide both training in HPA as well as time and space to work on written products. In addition, personal sessions with mentors during and outside the workshops provide support for protocol development, address specific data collection and analysis issues and offer guidance for paper writing. Each Fellow also receives a bursary to support themselves in working towards their PhD.

## Overview of Papers in This Edition

The seven papers presented in this special issue offer new perspectives and insights on particular experiences and dimensions of health policy change and health system development in LMICs. Four of these papers present empirical work – both drawing on established theoretical frames to deepen their inquiry and testing those frames through rich inductive analysis of experience. The other three present innovative qualitative synthesis papers founded on systematic literature reviews.

Unusually within the broader HPA literature four of these papers specifically address policy implementation issues. This focus may itself indicate the particular, but overlooked,^5^ importance of such issues within country settings, and the papers together illuminate the bottom-up forces shaping implementation.

Guinaran et al ^6^ and Ramani et al^7^ present empirical studies using different bodies of theory. Guinaran et al^6^ analyse the challenges faced in implementing the 2013 Joint Memorandum Circular on Indigenous People’s Health in the Philippines. Focusing explicitly on how power is practiced by actors located at different interfaces within a devolved health system, the analysis reveals both the hidden power of hierarchical and bureaucratic norms and the agency exercised by indigenous health managers. In the form of programme targets imposed down the system, the norms work against implementation by drawing implementers’ attention away from the indigenous health policy and towards existing vertical programmes. However, the agency derived from indigeneity, seen as a commitment to the policy, drove entrepreneurial managerial action that supported implementation. This analysis reveals the enduring dynamic between top-down and bottom-up power flows in implementation, through a deep inquiry around power that is sensitive to context and policy specificities. Ramani and colleagues’^7^ inquiry also reveals this dynamic in the setting of rural Indian primary healthcare facilities. It specifically draws on street level bureaucracy theory to investigate the coping behaviours of medical doctors working in these units, and what influences their exercise of this discretionary power. Amongst other factors, it shows again how targets established to drive vertical programmes work to undermine implementation – in this case, the provision of comprehensive primary healthcare services. This analysis is unusual within the limited LMIC literature on street level bureaucrats in its explicit focus on doctors, revealing the conflict between their professional and bureaucratic identities, and its detailed unpicking of coping strategies. Both papers, finally, point to the importance of the actors involved in policy implementation and to encouraging mid-level managers to be reflective about their leadership,^8^ in contrast to the common top-down approaches of donor- and central government-led implementation.

Derkyi-Kwarteng et al^9^ offer further insights into implementation experience in their qualitative synthesis of literature addressing the question: why do unintended out-of-pocket payments continue to be made for services covered by health insurance schemes in sub-Saharan Africa? The synthesis highlights, again, the exercise of frontline discretionary power as an explanation of such payments – and as a means of coping with conflicting policy objectives and resource constraints. However, there are also instances of corrupt practice. These may either be purely for personal gain or be part of a ‘corruption complex’ enabled by the institutional norms of the wider system, such as powerless citizens and weak accountability. Implementing financing reforms through top-down approaches is, then, judged likely to generate implementation gaps.

The final paper considering policy implementation, by Parashar et al,^10^ presents a methodological reflection around the application of interface analysis in investigating actors’ exercise of power in implementation. Purposefully seeking to bring the work of rural sociologist Norman Long^11^ to HPA audiences, the paper illuminates the key dimensions of interface analysis through an interpretive synthesis of a set of published studies. The paper both introduces Long’s thinking and presents reflections/findings from the synthesis around four key questions: the type of actor interfaces formed, the power practices observed, the effect of such power practices on implementation and the underpinning factors for the power practices. Importantly, the paper also provides guidance on how to conduct interface analysis, specifically unpacking the notion of actors’ lifeworlds as a framework for understanding the factors influencing actors’ exercises of their power. It adds to the very limited methodological guidance about how to conduct actor-centric power inquiries in LMIC settings, drawing on development theory.

Other policy stages are examined by Okeyo et al^12^ and Mukuru et al.^13^ Oeyo et al^12^ consider the policy adoption (formulation) process around the ‘first thousand days of childhood’ initiative, an intersectoral programme to improve child health outcomes, within one province of South Africa. Explicitly considering the ideas, interests and institutions influencing policy adoption, this paper considers what factors hinder moves towards policy implementation following apparently successful agenda setting processes. Like the implementation pieces already considered, it again highlights the challenges of implementing horizontal health policies that cut across organisational or bureaucratic silos given the dominant tendency to work vertically within a specific programme or sector. In this case, the horizontal policy was intended to be an intersectoral approach – but its initial conceptualisation as a health sector-specific initiative generated obstacles to its subsequent implementation across sectors. The analysis raises important questions about whether and how health sectors and systems can break free of historical ideas, interests and institutions to enable the intersectoral action recognised as essential in addressing the social determinants of health. Founded in policy elite theory, Mukuru et al^13^ present a detailed inquiry into how policy elites’ interests and power dynamics influenced decision-making around maternal health policy in Uganda. The paper considers political, state and donor actors working at national level, illuminating diverse driving interests including vote-winning, personal economic interests, pragmatic interests and altruistic interests. Providing detailed insights into the differences between policy-making guidance and what happened in practice, it argues that maternal health policy was shaped by powerful domestic elite groups who were generally more self-interested than altruistic. As a result, the policies did not fully address the problem of maternal health mortality or serve public interests.

Finally, Whyle and Olivier’s^14^ rich conceptual paper draws on policy analysis theory in considering the capacity of health systems to influence and generate social values. Through an interpretive synthesis of HPSR literature, founded on a qualitative systematic review, the paper generates new explanatory theory about how health systems generate social value. The paper’s policy implications address the types of policy-making processes needed to support health system action to promote health equity and wider social justice, and the role of policy-makers as interpreters of social values. Researchers, meanwhile, are challenged to adopt synthesis approaches that capture complexity, use language that recognises it, and generate policy-relevant conceptual insights.

Taken together these papers present valuable insights into different stages within health policy processes in various LMIC settings, as well as offering methodological and conceptual insights of importance both to future research and to health system development. The papers are grounded in their first authors’ deep understanding of the lived realities of policy change in their settings and enhanced by the theoretical frames on which they draw. As a group they illuminate an actor-centric approach to policy analysis, deepening understanding of how actors’ practices of power impact on policy change in LMICs, which is an acknowledged weakness of existing research.^5^ At the same time, they highlight features of health system contexts that limit actors’ influence over policy change and, in some cases, point to the ways in which broader societal factors and ideas are infused into these systems and into actors’ lifeworlds.

In sum, the papers included in this edition reveal the interaction between policy processes and health system complexity that is a core concern of HPSR, and illuminate the relevance of HPA to health system development. Together they also highlight how the HPA Fellowship programme is contributing to strengthening HPA capacity in LMICs.

## Ethical issues

Not applicable.

## Competing interests

Authors declare that they have no competing interests.

## Authors’ contributions

All authors conceptualised the piece. LG prepared the first draft. ZS and MSK reviewed and commented on the first draft. LG finalised the paper.

## Authors’ affiliations

^1^Health Policy and Systems Division, School of Public Health and Family Medicine, University of Cape Town, Cape Town, South Africa. ^2^Department of Global Health and Development, London School of Hygiene and Tropical Medicine, London, UK. ^3^The Alliance for Health Policy and Systems Research, World Health Organization, Geneva, Switzerland.
